# A Pilot Study of Aerosolization of Infectious Murine Norovirus in an Experimental Setup

**DOI:** 10.1007/s12560-024-09595-2

**Published:** 2024-05-02

**Authors:** Roderik Purhonen, Nina S. Atanasova, Julija Salokas, Jonathan Duplissy, Emil Loikkanen, Leena Maunula

**Affiliations:** 1 Department of Food Hygiene and Environmental Health, Faculty of Veterinary Medicine, University of Helsinki, Helsinki, Finland; 2 Molecular and Integrative Biosciences Research Programme, Faculty of Biological and Environmental Sciences, University of Helsinki, Helsinki, Finland; 3 Institute for Atmospheric and Earth System Research (INAR), Faculty of Science, University of Helsinki, Helsinki, Finland; 4 Atmospheric Composition Unit, Finnish Meteorological Institute, Helsinki, Finland

**Keywords:** Murine norovirus, Aerosol, Infectivity, Cell culture, Aerosolization

## Abstract

Human norovirus is transmitted mainly via the faecal-oral route, but norovirus disease outbreaks have been reported in which airborne transmission has been suggested as the only explanation. We used murine norovirus (MNV) as a surrogate for human norovirus to determine the aerosolization of infectious norovirus in an experimental setup. A 3-l air chamber system was used for aerosolization of MNV. Virus in solution (6 log_10_ TCID_50_/ml) was introduced into the nebulizer for generating aerosols and a RAW 264.7 cell dish without a lid was placed in the air chamber. Cell culture medium samples were taken from the dishes after the aerosol exposure time of 30 or 90 min, and the dishes were placed in a 37 °C, 5% CO_2_ incubator and inspected with a light microscope for viral cytopathic effects (CPEs). We determined both the infectious MNV TCID_50_ titre and used an RT-qPCR assay. During the experiments, virus infectivity remained stable for 30 and 90 min in the MNV solution in the nebulizer. Infectious MNV TCID_50_ values/ml of 2.89 ± 0.29 and 3.20 ± 0.49 log_10_ were measured in the chamber in RAW 264.7 cell dish media after the 30-min and 90-min exposure, respectively. The MNV RNA loads were 6.20 ± 0.24 and 6.93 ± 1.02 log_10_ genome copies/ml, respectively. Later, a typical MNV CPE appeared in the aerosol-exposed RAW cell dishes. We demonstrated that MNV was aerosolized and that it remained infectious in the experimental setup used. Further studies required for understanding the behaviour of MNV in aerosols can thus be performed.

## Introduction

Human noroviruses (HuNoVs) are the most common causes of epidemic and sporadic cases of acute gastroenteritis worldwide (Robilotti et al., 2015). They spread mainly by the faecal-oral route and are transmitted via food, water or contact with fomites. However, reports of outbreaks have appeared in which transmission routes other than airborne have been unlikely. For instance, during a hospital outbreak caused by HuNoV, Sawyer et al. (1988) determined that neither food nor water exposure could be identified, leaving the only option for exposure to being airborne particles. HuNoVs result in approximately 700 million infections and 200 000 deaths globally per year and cause symptoms that include diarrhoea, vomiting, and stomach pain (Graziano et al., 2019). So far, airborne transmission of HuNoVs has not been extensively studied. With the recent pandemic caused by severe acute respiratory syndrome coronavirus 2 (SARS-CoV-2), the importance of understanding airborne transmission of pathogens has become paramount.

HuNoV belongs to the *Caliciviridae* family of non-enveloped, positive-sense single-stranded RNA viruses (Graziano et al., 2019). HuNoV has a 7.5-kilobase-long genome and virus particles are approximately 27–30 nm in diameter.

The commonly used surrogate for HuNoV is murine norovirus (MNV), which also belongs to the *Norovirus* genus and morphologically resembles HuNoVs (Wobus et al., 2006). It can be cultivated to relatively high titres in a continuous laboratory cell line, whereas HuNoV is difficult to cultivate in vitro. HuNoVs are currently cultivated in human intestinal stem cell-derived enteroids (HIEs), which support the cultivation of several HuNoV strains (Ettayebi et al., 2016). However, difficulties remain in cultivating all the strains.

Previous aerosol studies have shown that HuNoVs in aerosols may be a relevant factor indoors regarding the spread of disease. One study investigated hospital rooms with HuNoV-infected patients by taking air samples (Alsved et al., 2020a). The study revealed that HuNoV RNA was found in air samples from 10 different patients with concentrations of airborne HuNoVs ranging from 5 to 215 copies/m^3^. Another study focused on the release of MNV aerosols during toilet flushing (Boles et al., 2021). This study used MNV-seeded toilet water with a scale of  10^5^– 10^6^ total RNA copies and managed to collect MNV aerosols in concentrations ranging from 383 to 684 RNA copies/m^3^. Similar results were seen in yet another study, in which air samples were taken from a hospital wastewater treatment plant showing HuNoV aerosol concentrations levels of 10^7^ counts/m^3^ air (Uhrbrand et al., 2017). Airborne HuNoV may pose a potential health risk indoors, although the risk may be low.

Laboratory experiments studying aerosolization of infectious viruses have been conducted not only with respiratory viruses, but also enteric viruses, such as rotaviruses (e.g. Sattar el al., 1984) and more recently with MNV (Alsved et al., 2020b; Bonifait et al., 2015). Creager et al. (2018) showed how cultured cells become infected after being exposed to aerosolized influenza virus. They used a jet nebulizer to generate the aerosols and then directed them into an exposure chamber with cultured cells or into a BioSampler collection device. Generally, other aerosol experiments conducted with MNV have used various kinds of nebulizing devices and collection methods. Bonifait et al. (2015) reported using a single-jet atomizer to produce aerosolized viruses and collected them with a cyclone aerosol sampler. Alsved et al. (2020b) reported using both an atomizer and a sparging liquid aerosol generator (SLAG) to produce aerosolized viruses and collected them with a BioSampler.

In this study, an Omron Comp A.I.R. Pro C900 jet nebulizer was used to create aerosols from a virus suspension. Jet nebulizers take compressed air/gas and use a venturi to cause a pressure differential that draws liquid from the reservoir to the gas (Hyers et al., 2021). This causes the liquid to be broken apart into a spray. Jet nebulizers produce a larger variety of various size aerosols than ultrasonic nebulizers, which produce aerosols of more uniform particle size. Therefore, the jet nebulizer’s larger particle variety mimics natural aerosol generation better than the ultrasonic nebulizer.

The aim of this pilot study was to establish a simple aerosolization system that could enable studies using infectious MNV in aerosols. We tested the ability of aerosolized virus to infect RAW 264.7 cells, using a cell-containing dish positioned in the air chamber to reveal the infectivity of the virus in aerosols. In addition, we compared the infectious virus loads and the RNA levels in the samples taken from the cell culture media in the dish after the 30-min or 90-min aerosol exposures.

## Materials and Methods

### Murine Norovirus and Preparation of the Virus Stock

We performed aerosolization experiments, using cultivable murine norovirus (strain MNV-1) obtained from Dr. Herbert W. Virgin at the Washington University School of Medicine (St. Louis, MO, USA). MNV was cultured in RAW 264.7 cell line (ATCC® CRL2278™) grown in Dulbecco’s modified Eagle’s medium (DMEM; Gibco), containing 10% foetal bovine serum (FBS, Gibco) and 1% glutamine–penicillin–streptomycin (Sigma-Aldrich). The cells were kept at 37 °C in a 5% carbon dioxide (CO_2_) atmosphere and maintained by producing new subcultures every 2–3 days. Cells from passage 12–30 were used for the experiments.

To produce virus stock, MNV was inoculated into a 70% semiconfluent cell monolayer and cultivated for 41 h, after which the infected cells were frozen and thawed 3 times to release the viruses.

To remove DMEM’s growth solution proteins from the virus stock, the supernatant was subjected to ultrafiltration (Amicon Ultra 100 K, Merck Millipore, Cork, Ireland) at 3200 × g, for 35 min at + 4 °C. The remaining supernatant was recovered and diluted with phosphate-buffered saline (PBS) 1:10. The titre of the diluted virus stock was determined to be approximately 10^6^ (6 log_10_ units) (50% tissue culture infectious dose TCID_50_)/ml with RNA content of 10^10^ (10 log_10_) genome copies (gc). It was stored at − 70 °C. Antifoam A concentrate (Sigma-Aldrich, St. Louis, MO, USA) was added to the virus solution (30 µl into 10 ml of solution) before the aerosol experiment was conducted to prevent foaming in the nebulizer.

### Experimental Aerosolization Setup

**Fig. 1:**
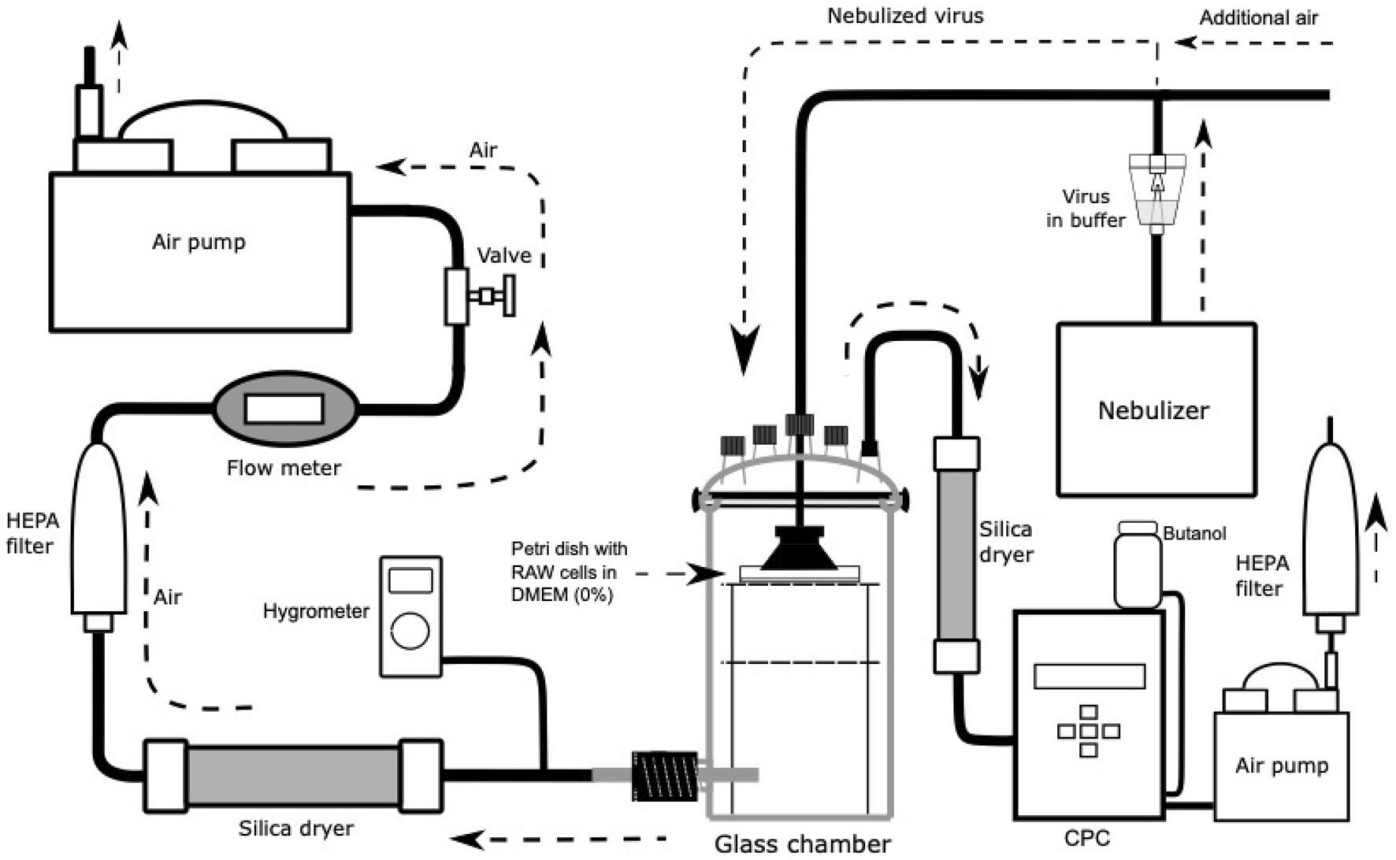
Schematic representation of the aerosol chamber and sample collection. *HEPA* high-efficiency particulate air filter, *DMEM* Dulbecco’s modified Eagle’s medium, *CPC* condensation particle counter

An experimental setup for aerosolization and collection of viruses was used to study the aerosolization of MNV (Fig. 1). An Omron CompAir Pro NE-C900 nebulizer and Charles Austen Pumps Ltd. (model B105; Surrey, England) were used to generate a consistent flow of virus aerosols. The nebulizer produces an airflow of virus aerosols at 8.8 L per minute (lpm), and the pump attached to the system takes 10 lpm, while an additional 1.2 lpm is taken from a separate split vent. This split in the airflow was implemented to reduce humidity in the chamber during prolonged measurements.

The airflow of the virus aerosols was directed into a 3-l autoclavable, sealed glass chamber through a metal funnel that directed the aerosols onto a RAW 264.7 cell dish (9 cm in diameter) with 4 ml of 0% DMEM as collection liquid. Employing an impaction sampling method, the medium surface of the cell dish collects the viruses. The air chamber was based on a previously developed model (Sofieva et al., 2022). The glass cylinder and the lid of the chamber were manufactured by Laborexin Corporation (Helsinki, Finland), while the inner metal scaffold was constructed by Clean Touch Medical (Helsinki). The insulating gaskets for the lid were made by Etra Oy (Helsinki), a division of the Etola group (Helsinki). After passing through the chamber, the airflow was dehumidified in a desiccator to protect the high-efficiency particulate air (HEPA) filter and flow meter from excessive moisture. The relative humidity (RH) of the system was measured with a hygrometer (Humicap, Vaisala) and varied at 90 –95%. Experiments were performed inside a safety cabinet. The temperature of the laboratory was 22.8 ± 0.8 °C.

### Experiment Description

**Fig. 2:**
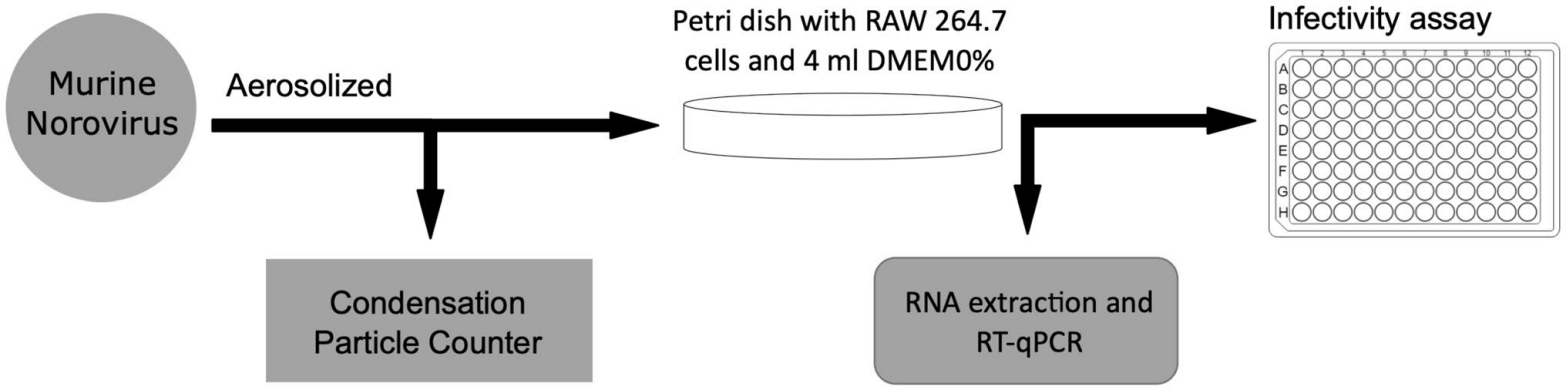
Experimental workflow chart. *RT-qPCR* real-time quantitative reverse transcription polymerase chain reaction, *DMEM* Dulbecco Modified Eagle’s Medium

Figure 2 shows the principal steps of the aerosol experiments. The experiments were divided into three parts: a 15-min control experiment, followed by a 30-min and 90-min experiments. The aerosol experiment was initiated with the 15-min control experiment in which PBS was used in the nebulizer instead of virus solution to check the purity of the system. For the 30-min experiment, the PBS in the nebulizer was exchanged for a virus solution (approx. 10^6^ TCID_50_/ml, i.e. 6 log_10_ TCID_50_/ml), and a new RAW 264.7 cell dish was placed in the chamber. Then, the aerosols were collected on the cell dish for 30 min. Finally, for the 90-min experiment, the RAW cell dish was exchanged for a new dish, and the virus aerosols were collected for 90 min. The nebulizer could only hold 7 ml of liquid and consumed approximately 2 ml/15 min of virus solution. Therefore, 2 ml of new virus solution was introduced into the nebulizer during the aerosol experiment at 15-min intervals.

Samples were taken of the medium in the cell dishes after the experiment and of the nebulizer’s virus solution before and after the experiments, the latter once a day. After the samples were taken, the cell dishes from the 30-min experiment and PBS control experiment were placed in incubation for 30 min at 37 °C in 5% CO_2_ to allow more time for the viruses to infect the cells. This incubation was not done for the 90-min experiment cell dish. Next, fresh 10% DMEM medium was added to the cell dishes, which were thereafter incubated for 3–4 days, inspected for viral cytopathic effects (CPEs) with a light microscope, and a portion of the medium was frozen at − 70 °C for RNA extraction. The RNA levels were determined after five experiments.

### ***TCID***_***50***_*** Assay***

The infectious viral loads in the samples were measured in 96-cell plates by TCID_50_ test (Mosselhy et al., 2022). The plates were prepared prior to the aerosol experiment by adding 200 µl of 10% DMEM containing approximately 4 log_10_ units of RAW 264.7 cells per well and incubating them for 3 days at 37 °C in 5% CO_2_. Firstly, serial tenfold dilutions were made with the MNV samples from the aerosol experiment. Next, 200 µl of cell culture medium was removed from the cell culture plate before adding 100 µl of fresh 10% DMEM and 100 µl of diluted or original sample per well. Six parallel wells were used for each sample and dilution. The plates were incubated for 1 h at 37 °C in 5% CO_2_, after which the inoculates were replaced by 200 µl 10% DMEM per well. The plates were then incubated at 37 °C in 5% CO_2_ for 5 days. The plates were inspected throughout the week for viral CPE with a light microscope and marked as positive or negative. After the final reading on the 5th day, the TCID_50_/ml value was calculated with the Spearman–Kärber algorithm, using a TCID_50_ calculator (Hierholzer & Killington, 1996). The limit of detection was 0.5 log_10_ TCID_50_/ml.

### RNA Extraction and RT-qPCR

RNA extraction was performed from samples stored frozen at − 70 °C with the E.Z.N.A.® RNA isolation kit according to the manufacturer’s instructions, using 150 µl as a sample volume and 50 µl as an elution volume. The MNV RNA levels were determined by a real-time quantitative RT (reverse transcription)-polymerase chain reaction (RT-qPCR), using specific primers and a FAM-labelled probe. We used the Qiagen QuantiTect Probe RT-PCR kit (Qiagen, Hilden, Germany) with primers MNVfor and MNVrev, and a probe MNVproH designed by Hewitt et al. (2009). A 20-µl reaction, including 5 µl of sample RNA, was cycled by a Rotor-Gene 3000A (Corbett Research) according to Rönnqvist et al. (2014). Negative controls, PBS and water, were included for the RNA extraction and PCR, respectively. The sample cycle threshold (ct) values were plotted to a standard curve that was created, based on the results obtained from a tenfold dilution series made from the MNV RNA of stock virus, revealing the end point. The theoretical limit of detection for the RNA concentration in the original sample was 1.8 log_10_ gc/ml.

### Condensation Particle Counter (CPC)

Aerosol particle number concentration was measured using a condensation particle counter (CPC 3772, TSI). Inside the CPC, aerosol particles larger than 10 nm are activated with saturated butanol vapour, and then further grow to a size that can be detected by the in-built optical particle counter (OPC). The CPC measures the total number of all particles bigger than 10 nm entering the CPC, with 1 s-time resolution. It has a 1 l/min intake airflow. A silica dryer was installed at the entrance of the CPC to avoid high humidity entering the CPC, which could have otherwise reduced the detection efficiency of the CPC.

### Statistics

The experiments were repeated 6 times. For TCID_50_ assay, each dilution was added to six parallel wells. RNA values were obtained by running all samples in RT-qPCR twice. The values were used in calculations after they were transformed to log_10_. We used Excel in calculations and GraphPad Prism Software (version 10.2.0) to create the graphs. We used SPSS (IBM) for statistical analyses (Welch test). *p* value of 0.05 was considered as threshold of significance.

## Results

### Virus Preserved Viability in the Nebulizer; Preliminary Tests

To reduce foaming of the virus suspension in the nebulizer during the experiments, we added antifoam A reagent into the suspension before initiation of the aerosolization experiment. We optimised the amount of the reagent before starting the main experiments and confirmed that the infectivity of the virus was not affected by it.

We used PBS solution instead of virus solution as a negative control for aerosol production and as proof of cleanness of the tubing systems (done once per experiment day). These negative controls remained negative in all experiments when tested with the RT-qPCR.

**Fig. 3:**
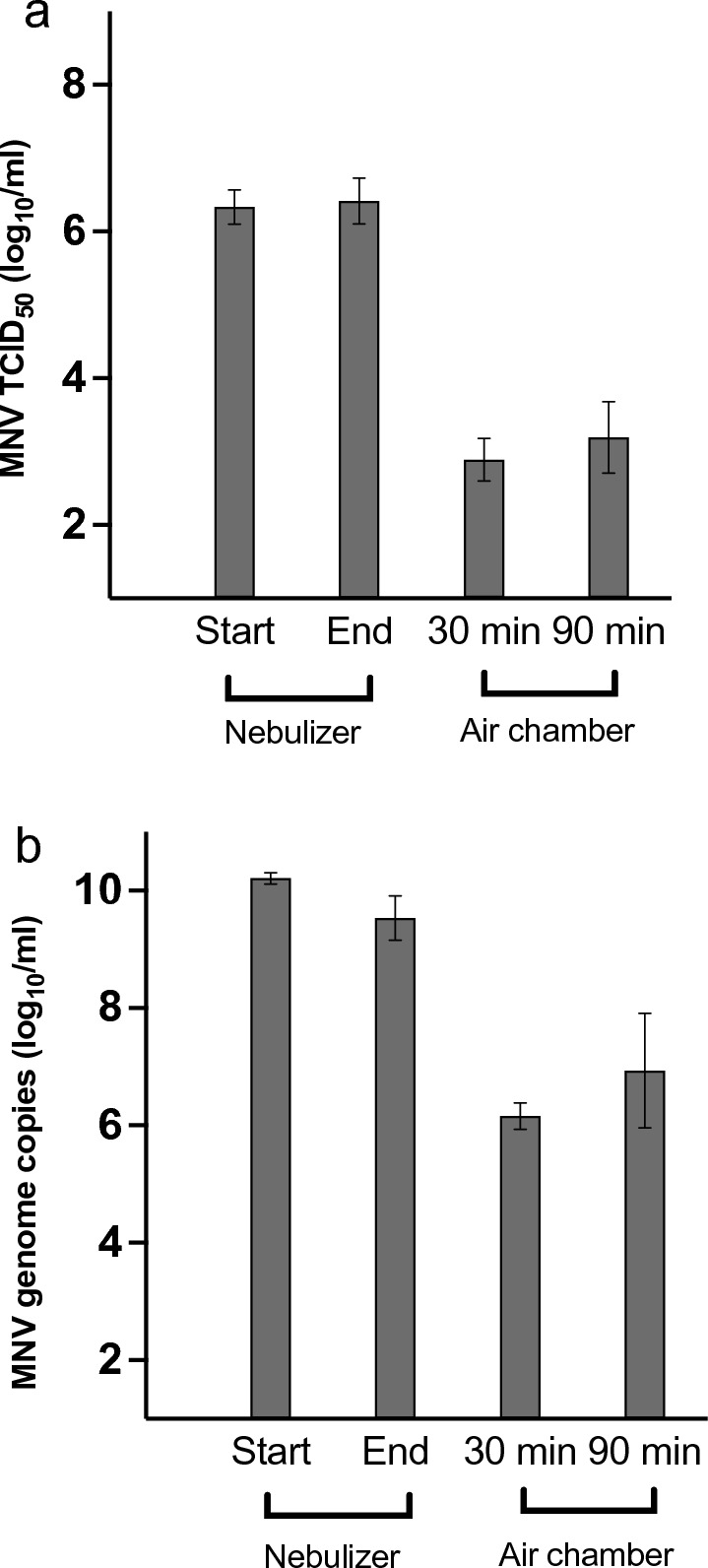
MNV infectious virus concentrations and genome copies in the original virus solution in the nebulizer (experiment start and end points) and in liquid of the cell dish after 30-min and 90-min aerosol exposure in the air chamber. **a**: infectious MNV, **b**: MNV RNA levels. Std deviations are indicated. *MNV* murine norovirus, *TCID*_*50*_ 50% tissue culture infectious dose

During the aerosol experiments performed at room temperature, virus infectivity remained relatively stable for 30 min and 90 min in the nebulizer MNV solution (experiment start and end points 6.33 ± 0.24 vs. 6.42 ± 0.31 log_10_ TCID_50_/ml, respectively; *p* > 0.05; Fig. 3a). As the standard deviations reveal, the results of the MNV TCID_50_ titres at the start of each experiment were reproducible, but we observed more variation in the virus concentration of the solution in the nebulizer at the end of the experiment (for the RNA values, see below).

### Aerosolized MNV Infected RAW 264.7 Cells Placed in the Air Chamber

CPC measurements confirmed the presence of high numbers of nebulized aerosol particles (~ 65,000 particles per cm^3^) in the air inside the glass chamber. This number of aerosol particles combines nebulized virus particles and other nebulized aerosol particles from the solution. Unfortunately, further systematic analysis of the aerosols with the CPC became impossible, due to recurrent obstruction of the OPC inside the CPC during each experiment.

After incubation at 37 °C in 5% CO_2_ for 3–4 days of the RAW cell dishes exposed to aerosols for 30 min in the air chamber, a typical MNV CPE for the RAW cells became apparent. We observed high numbers of genome copies (11 log_10_ gc/ml) when aliquots of the cell culture liquid from the dish were subjected to RT-qPCR at this stage, demonstrating that the virus had propagated in the cells. RAW cell dishes exposed to PBS-containing aerosols expressed no CPEs after incubation and, as mentioned earlier, the medium remained negative in the RT-qPCR assay.

### Murine Norovirus Preserved Its Infectivity During the Aerosolization Experiments

We sampled cell medium from the dish in the air chamber after the selected time periods of aerosolized MNV exposure and measured the viral loads in 96-well RAW cell plates, using serial tenfold dilutions. The infectious MNV TCID_50_ values/ml measured from the dish medium in the air chamber were 2.89 ± 0.29 and 3.20 ± 0.49 log_10_ after 30 min and 90 min, respectively (*p* > 0.05; Fig. 3a). Further detail revealed that the average titres for the 90-min samples were close to 3 times the result from the 30-min experiments, as would be expected.

Regarding the concentrations of viable virus in the nebulizer compared with those in the medium samples taken from the RAW cell dish in the air chamber, the TCID_50_ values clearly differed both in the 30-min and in the 90-min experiments, showing a difference of 3.44 ± 0.27 and 3.14 ± 0.57 log_10_ units/ml, respectively.

We also performed once an experiment in which we showed that further dilutions of 1:10 and 1:20 diluted virus solution (final MNV stock dilution 1:100 and 1:200) in the nebulizer still resulted in virus growth on a RAW cell plate in the air chamber after 30 min, and the medium samples showed the presence of infectious virus (1:100 dilution, 0.7 log_10_ TCID_50_/ml; 1:200 dilution, 0.7 log_10_ TCID_50_/ml) and viral RNA (1:100 dilution, 3.6 log_10_ gc/ml; 1:200 dilution, 3.5 log_10_ gc/ml).

### RT-qPCR Reveals the Total Amount of Viral RNA from Infectious and Non-infectious Viruses

The RNA values of MNV in the nebulizer determined with RT-qPCR decreased during the experiments (at the start 10.2 ± 0.10 log_10_ gc/ml vs. in the end 9.53 ± 0.41 log_10_ gc/ml; *p* < 0.05; Fig. 3b**)**, unlike the trend in the infectious virus experiments above. The MNV RNA values measured from the dish media in the air chamber were 6.20 ± 0.24 and 6.93 ± 1.02 log_10_ gc/ml after 30 min and 90 min, respectively (*p* < 0.05). As in the viability test, we observed more variation in the viral RNA loads in the 90-min experiments than in the 30-min experiments (std deviations 1.02 vs. 0.24 log_10_).

The MNV RNA loads from the same medium samples as measured by TCID_50_ assay for the infectious virus above were also several log_10_ units lower than those in the virus solution in the nebulizer; the difference was 4.0 ± 0.22 log_10_ gc/ml and 3.3 ± 1.02 log_10_ gc/ml for the 30-min and 90-min experiments, respectively **(**Fig. 3b**)**.

## Discussion

We were able to establish a relatively simple aerosolization system that can be used to study the fate and behaviour of infectious MNVs when aerosolized in the laboratory. This is crucially important since we still need more data on how environmental conditions affect the infectivity of aerosolized viruses and on the ability of viruses to preserve their infectivity in air to tackle the spread of viruses indoors (Alsved et al., 2020b). We showed that MNV particles in aerosols preserved their infectivity, at least for some time. MNV in aerosols infected predisposed cells directly in the air chamber. In addition, infectious viruses were detected after the 30-min and 90-min aerosolization exposure times in the cell culture media. The infectivity and RNA results showed a similar tendency for the viral load to increase as a function of time when the 30-min and 90-min results were used, although these results were not as reproducible for infectivity as for viral RNA.

Bacteriophages as surrogates are ideal in that it is possible to perform experiments even outside the laboratory rapidly and economically and to prepare extremely high-titre virus stocks (Oksanen et al., 2022a; Salokas et al., 2023). This study, performed with a HuNoV surrogate, increased our experience in handling aerosolized non-enveloped viruses. It may also reveal some aspects that can help us understand the behaviour of aerosolized respiratory viruses, such as SARS-CoV-2, since cell culture is a commonly used platform for revealing the infectivity of all mammalian viruses. Enteric viruses, such as HuNoV, have the benefit of being very stable in the environment (Kotwal & Cannon, 2014), and this characteristic may be useful in the establishment of the system in comparison to less resistant enveloped viruses. Bonifait et al. (2015) demonstrated the stability of MNV in the nebulizer during the aerosolization experiments. Our results in this study are in line with those of Bonifait et al. (2015). We should, however, bear in mind that a surrogate virus almost never gives results identical to those of the human virus. For instance, Belser et al. (2022) demonstrated that there can be variance in stability even among different influenza virus strains.

Previously, Creager et al. (2017) used a similar aerosolization setup for influenza viruses. The differences were that they used a cell plate with a membrane under an air–liquid interface suitable for respiratory viruses, while we cultivated cells as a monolayer in our study. Creager et al. (2018) have later published a thorough article describing the details of their system. As Creager et al. (2018), we also placed the cells in the air chamber, but we used a high RH reported suitable for non-enveloped viruses (polio) in a review by Yang and Marr (2012). We performed these experiments at room temperature according to most studies. Earlier, airborne rotavirus survival was found enhanced at lower temperature of 6 °C depending on RH (Ijaz et al., 1985), whereas more studies are still required regarding the survival of aerosolized viruses in tropical climate.

Detection of infectious viruses in indoor air aerosols is challenging. In our study, we found that the traditional cell culture method for MNV worked well. Alsved et al. (2020b) also used MNV in an aerosolization study with a slightly different detection method. Virus was propagated in RAW cells for 1 day, after which researchers used negative-sense RNA detection by RT-qPCR to determine the RNA load in the cells. In another study of Bonifait et al. (2015), the researchers did not use virus cell culture for virus detection, but rather propidium monoazide (PMA)-RT-qPCR, measuring RNA from potentially infectious and disintegrated virus particles. These kinds of chelating reagent methods linked to genome detection can be used for some purposes, but in some settings, they may result in overestimation of the persistence of viruses, although they are more reliable than genome detection by (RT-)PCR only (Parshionikar et al., 2010; Stobnicka-Kupiec et al., 2022).

A high-titre virus was used when aerosols were generated, in line with most studies. Alsved et al. (2020b) used MNV of approximately 10^6^ TCID_50_/ml and Bonifait et al. (2015) 10^7^ plaque-forming units (PFUs)/ml, the concentrations of which were comparable to the concentration of virus we used. In some real-world situations, such as during vomiting, high numbers of HuNoVs can be aerosolized temporarily in addition to droplets, since vomit can contain 10^7^ gc/ml HuNoV RNA (Yezli & Otter, 2011). A restaurant outbreak reported by Marks et al. (2000) revealed an inverse relationship in attack rates for the persons infected with the distance from a person who vomited in the same space, although spread through fomites could not be excluded (Xiao et al., 2017). Workers in wastewater treatment plants may also be exposed to high levels of viruses and other microbes aerosolized in air.

In our study, it was not possible to accurately compare the total quantities of infectious viruses in the nebulizer and in the air chamber, due to the time factor in the continuous system. However, infectious virus concentrations were clearly low or lower in the air chamber, which hardly renders them detectable in the viability test techniques currently used in indoor air. Similar observations have been made by other investigators. For instance, Salokas et al. (2023) showed that the number of viable Phi6 bacteriophages was 10^6^–10^7^ PFUs/ml, whereas about 10^10^ PFUs/ml Phi6 virus was used in the nebulizers. Alsved et al. (2020b) observed a total difference of 5 log_10_ units in the start solution and the collection liquid, which is more than the total difference of approx. 3.5 log_10_ units in our study, which may have resulted from the aerosols not being dried in our study. Drying of virus-containing aerosols causes even harsher conditions for viruses, thus leading to greater dilutions (Alsved et al., 2020b). So far, we have lacked techniques sensitive enough to detect infectious virus levels in air under real-world conditions (Oksanen et al., 2022b). Future efforts should focus on developing these sensitive techniques.

In the real world, measuring low amounts of viruses in indoor air is thus often successful only when RNA-based detection methods are used, which cannot reveal the presence of infectious viruses. In the case of HuNoV, all virus detection is still mainly based on genome detection. In our study, unlike infectious virus amounts, the RNA counts were also high in the air chamber. The difference between measured infectious virus and RNA levels may have resulted from the high proportions of RNA and non-infectious virus particles in the virus stock, which qPCR can detect. Viruses also lose some of their infectivity when they are aerosolized; a loss as high as 10^4^–10^5^ was reported for influenza virus by Brown et al. (2015). In our study, viruses probably survived better once they had settled onto the medium in the cell dish than in the air of the chamber. On the other hand, portions of the viruses were likely already attached to the cells at the bottom of the dish during the aerosol exposure and thus were no longer detectable from the medium by the infectious assay.

The chemical compositions (inorganic salts, metals, carbonaceous compounds, and water) in atmospheric aerosols likely impact how viruses persist in them and how aerosols behave in the air, but the amount of data available remains limited (Ahlawat et al., 2022). We added antifoam A, which suppresses foaming of the virus solution in the nebulizer according to Verreault et al. (2015), although the authors used it for experimental aerosolization of bacteriophages. Although our preliminary testing revealed no other changes in our system with or without antifoam. A addition, our study likewise does not reveal whether the use of antifoam A could have increased the number of aerosols or affected their size in the air chamber. Further testing is required to determine the answers.

This study has its limitations but remained above all a simple setup that worked well in our hands. These kinds of studies require an interdisciplinary working team, including those with expertise in virology, aerosol science and atmospheric science. Our study did not probe more deeply into the nature of these aerosols, and no ageing of the aerosols was done. Some of the parameters, such as humidity, were at the levels not normally used indoors. In Verreault et al. (2015), surrogate MS2 survived best in the aerosols at RH 20%, but also performed well at RH 80%, (RH 50% in between), although the experiments were performed differently: the time points were 6 h and 14 h and an air drum was used. Future investigations will hopefully enable us to determine the limits for all these and other parameters.

## Conclusion and Future Aspects

This study does not reveal whether HuNoV can be transmitted via the atmosphere or not, but we showed that a surrogate virus, MNV, could be aerosolized and that the viruses remained infectious in the aerosols for some time in the experimental setting used. Thus, it would be worthwhile to probe more deeply into this mode of transmission.
